# Solidification Behavior of Polymer Solution during Membrane Preparation by Thermally Induced Phase Separation

**DOI:** 10.3390/membranes4010113

**Published:** 2014-02-28

**Authors:** Toru Ishigami, Yoko Nii, Yoshikage Ohmukai, Saeid Rajabzadeh, Hideto Matsuyama

**Affiliations:** Center for Membrane and Film Technology, Department of Chemical Engineering, Kobe University, 1-1 Rokkodai, Nada-ku, Kobe 657-8501, Japan; E-Mails: ishigami@port.kobe-u.ac.jp (T.I.); yoko2145bbc@yahoo.co.jp (Y.N.); y.ohmukai@gmail.com (Y.O.); rajabzadehk@people.kobe-u.ac.jp (S.R.)

**Keywords:** solidification rate, membrane preparation process, thermally induced phase separation, poly(vinylidene fluoride)

## Abstract

The solidification behavior of poly(vinylidene fluoride) (PVDF) solution during membrane preparation by thermally induced phase separation (TIPS) was investigated. Apparatus newly developed in our laboratory was used to quantitatively measure membrane stiffness during phase separation. In this apparatus, a cooling polymer solution, placed on a stage, is moved upwards and the surface of the polymer solution contacts a sphere attached to the tip of a needle. The displacement of a blade spring attached to the needle is then measured by a laser displacement sensor. Different phase separation modes, such as liquid-liquid (L-L) phase separation and solid-liquid (S-L) phase separation (polymer crystallization) were investigated. In the case of S-L phase separation, the stiffness of the solution surface began to increase significantly just before termination of crystallization. In contrast, L-L phase separation delayed solidification of the solution. This was because mutual contact of the spherulites was obstructed by droplets of polymer-lean phase formed during L-L phase separation. Thus, the solidification rate was slower for the L-L phase separation system than for the S-L phase separation system.

## 1. Introduction

**Figure 1:**
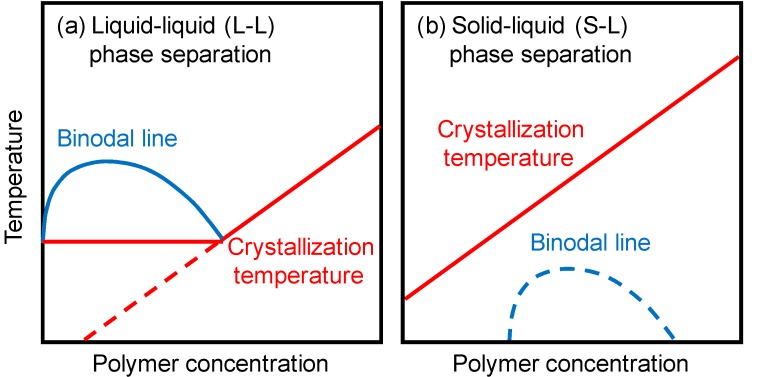
Phase diagrams in the thermally induced phase separation (TIPS) method. (**a**) Liquid-liquid (L-L) phase separation; (**b**) Solid-liquid (S-L) phase separation.

Polymeric porous membranes are generally prepared by phase separation of polymer solutions [1]. Phase separation can be induced by cooling or by the presence of nonsolvent. The former is thermally induced phase separation (TIPS), while the latter is nonsolvent induced phase separation (NIPS). In membrane preparation by TIPS, a polymer is dissolved in a diluent at high temperature, and then the homogeneous polymer solution is cooled to induce the phase separation. After the polymer is solidified by crystallization or glass transition, the diluent is extracted by solvent exchange and the extractant is usually evaporated to yield a microporous structure. There are two main types of phase separation for the crystalline polymers usually used in the TIPS process. When the binodal line, which is the border between homogeneous solution and phase-separated solution, is located above the crystallization temperature, liquid-liquid (L-L) phase separation occurs. The phase diagram in this case is shown in Figure 1a. The solution separates into a polymer-rich continuous phase and a polymer-lean droplet phase. On the other hand, if the crystallization temperature is higher than the binodal line, solid-liquid (S-L) phase separation (*i.e.*, polymer crystallization) occurs (Figure 1b). Many fundamental thermodynamic and kinetic studies have been done on membrane formation via TIPS [2,3,4,5,6,7,8,9,10].

A large number of microporous membranes with a variety of morphologies have been prepared from various polymers, including poly(vinylidene fluoride) (PVDF) [11,12], polyethylene [13,14], polypropylene [15,16], poly(methyl methacrylate) [17,18], polystyrene [19] and poly(ethylene-co-vinyl alcohol) [20,21], by TIPS. In particular, PVDF, which is a semi-crystalline polymer, has wide applications in manufacture of microfiltration (MF) and ultrafiltration (UF) membranes because of its excellent properties such as good mechanical strength, stability against aggressive chemicals and good thermal stability. PVDF microfiltration membranes prepared by the TIPS method have been commercially applied in drinking-water production and wastewater treatment.

Many studies of porous polymer membranes have focused on the prevention of membrane fouling and on improvement of membrane performance (such as solute rejection and water permeability), which directly affect the efficiency of water-treatment processes. Although the solidification behavior of a polymer solution during the phase separation process significantly affects the membrane preparation process, very few studies have reported solidification behavior of a polymer solution during the phase separation process. Understanding the solidification behavior of the polymer solution allows one to optimize the take-up speed of membrane preparation and the design of the coagulation (cooling) bath and rollers. Bonyadi* et al.* [22] and Yin* et al.* [23] indicated that the solidification rate affects the morphology of hollow fiber membranes prepared by NIPS.

In our previous study, we developed a new apparatus for directly measuring membrane stiffness and quantitatively analyzed the solidification behavior of polymer solutions during the NIPS process [24]. The effects of polymer concentration, composition of the coagulant, molecular weight of polymer and additives were investigated using this apparatus. Addition of hydrophilic additives to the polymer solution significantly accelerated the solidification rate because the uptake of water into the polymer solution was enhanced.

The aim of the present study was to investigate the solidification characteristics of polymer solutions during the TIPS process using this apparatus. Furthermore, we attempted to clarify the correlations between the solidification and crystallization behaviors of polymer solutions. The effects of phase separation patterns such as S-L and L-L phase separations on the solidification characteristics were investigated. The polymer examined was PVDF, chosen because PVDF membranes have been widely used commercially, as described above. As far as we know, this is the first work on the solidification characteristics of polymer solutions during the TIPS process.

## 2. Results and Discussion

### 2.1. Phase Separation Behavior

**Figure 2:**
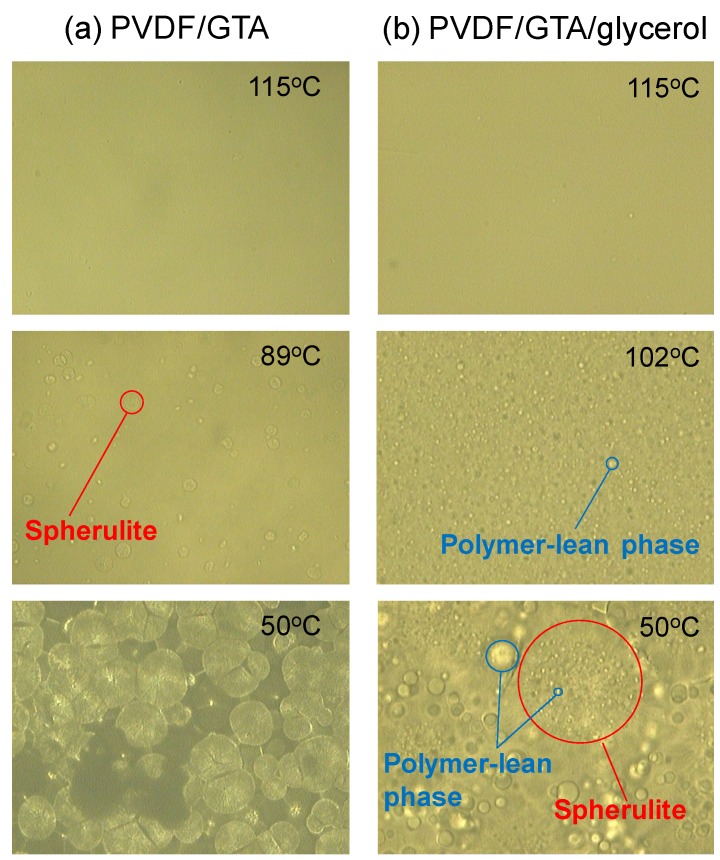
Optical micrographs during phase separation. (**a**) poly(vinylidene fluoride) (PVDF) (30 wt%)/glycerol triacetate (GTA) (70 wt%) system; (**b**) PVDF (30 wt %)/GTA (60 wt %)/glycerol (10 wt %) system.

Figure 2a shows optical micrographs of a PVDF (30 wt %)/Glycerol triacetate (GTA) (70 wt %) solution at various temperatures in the cooling process. Spherulite formation is clearly observed. In this system, the compatibility between PVDF and GTA is high, which results in a shift of the binodal line to below the crystallization temperature. Thus, polymer crystallization (S-L phase separation) occurs [25]. The micrographs show that isolated spherulites formed first, and then grew before finally contacting each other. Similar S-L phase separation was observed for the PVDF (30 wt %)/diethyl phthalate (DEP) (70 wt %) system. The dynamic crystallization temperatures (*T*_c_), at a cooling rate of 10 °C/min, for PVDF (30 wt %)/GTA (70 wt %) and PVDF (30 wt %)/DEP (70 wt %) solutions were 89 °C and 100 °C, respectively. The solubility parameters of PVDF [26], GTA and DEP [27] are 23.2 MPa^0.5^, 22.0 MPa^0.5^ and 20.4 MPa^0.5^, respectively. The solubility parameter of GTA is closer than that of DEP to that of PVDF. Therefore, the compatibility between PVDF and GTA is higher. When the compatibility is high, the crystallization temperature of the polymer solution decreases [3]. Thus, a lower crystallization temperature was obtained for the PVDF/GTA system.

The phase separation behavior of the PVDF (30 wt %)/GTA (60 wt %)/glycerol (10 wt %) system is shown in Figure 2b. Many droplets of polymer-lean phase were firstly formed by L-L phase separation. This is because the addition of glycerol (nonsolvent) lowered the compatibility between the PVDF and the solvent mixture, and shifted the binodal line to temperatures above the crystallization temperature [25]. The solubility parameter of glycerol is 36.2 MPa^0.5^ [27], which is far from that of PVDF (23.2 MPa^0.5^) [26]; glycerol is a nonsolvent for PVDF. Even in this system, spherulites formed later and grew.

### 2.2. Solidification Behavior of Polymer Solution during TIPS Process

**Figure 3:**
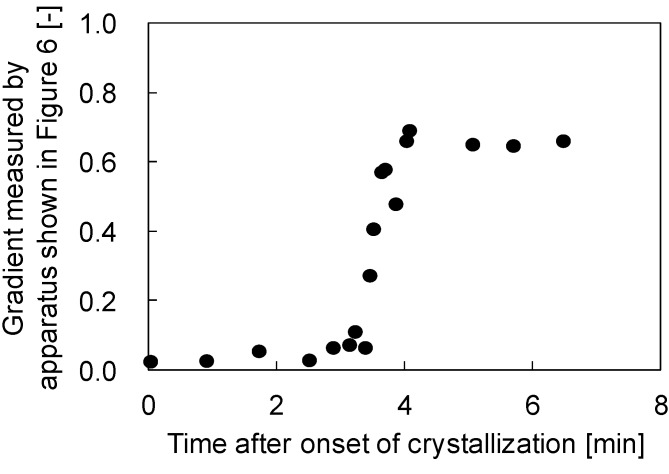
Variation of the ratio of the needle displacement to stage displacement with time after the onset of crystallization in the PVDF (30 wt %)/GTA (70 wt %) system.

Figure 3 shows the gradients of the plots of needle displacement against stage displacement *versus* time after the onset of crystallization. In this experiment, a PVDF (30 wt %)/GTA (70 wt %) solution was used. In the beginning, just after the onset of the crystallization, the gradients were almost zero. This means that the polymer solution was still soft and the sphere attached to the tip of the needle could easily penetrate into the polymer solution. This is reasonable, because in the initial stages of crystallization, the formed spherulites are dispersed in the solution and are not in contact with each other, as shown in Figure 2a; therefore the surface of the polymer solution is not so hard. After about 3 min, the gradient increased sharply before reaching a constant value. This increase in gradient probably begins when the spherulites begin to contact each other. After the spherulites were completely in contact, the stiffness of the solution surface became constant and the gradients also showed a constant value. The final constant gradient value obtained in this case was about 0.65; this value is lower than unity, suggesting that the solution surface was not completely hardened, even after the termination of crystallization.

**Figure 4:**
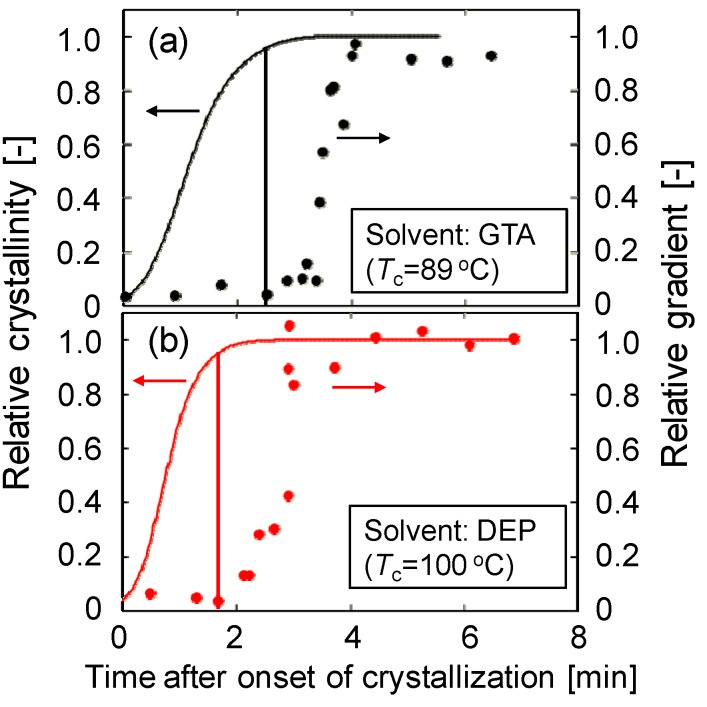
Comparison between relative gradient and relative crystallinity. (**a**) PVDF (30 wt %)/GTA (70 wt %) system; (**b**) PVDF (30 wt %)/DEP (70 wt %) system.

The comparison between the relative gradient and the relative crystallinity obtained by differential scanning calorimetry (DSC) is shown in Figure 4a. The relative gradient and crystallinity were normalized to the final gradient and crystallinity, respectively. The relative crystallinity shows a sigmoidal curve. In each figure, the time for the start of the gradient increase is shown by a vertical line. This time corresponds to the time just before the relative crystallinity became unity. This shows the stiffness of the solution surface started to increase significantly just before the termination of crystallization, that is, when mutual contact of the spherulites occurred. Thus, the result obtained in this work clearly indicates that the time the crystallization starts is not important for generation of stiffness of a phase separating solution; instead, the time when crystallization ends determines the solution stiffness.

Figure 4b shows the time courses of relative gradient and relative crystallinity for the PVDF/DEP system. As described above, the dynamic crystallization temperature (100 °C) of PVDF (30 wt %)/DEP (70 wt %) solution was higher than that (89 °C) of PVDF (30 wt %)/GTA (70 wt %) solution. Comparing Figure 4a,b, the relative crystallinity reached its final value at around 3.5 min after onset of crystallization in the case of the PVDF/GTA system, while the relative crystallinity reached at 2.5 min in the case of the PVDF/DEP system, explaining that the crystallization occurred more rapidly in the PVDF/DEP system than in the PVDF/GTA system. This is because the polymer chain movement is higher as a result of the higher crystallization temperature. Although the crystallization temperature was different, the same conclusion—that the stiffness of the solution surface began to increase significantly just before the termination of crystallization—was also obtained for the PVDF/DEP system. Thus, this conclusion is probably valid for all S-L type phase separation systems.

**Figure 5:**
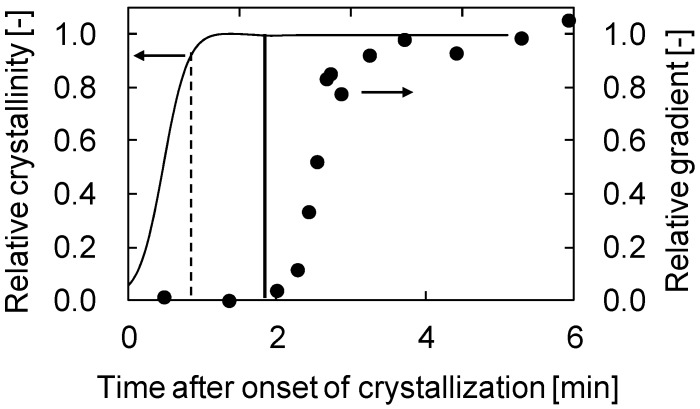
Comparison between relative gradient and relative crystallinity in PVDF (30 wt %)/GTA (60 wt %)/glycerol (10 wt %) system.

Figure 5 shows the results for the PVDF (30 wt %)/GTA (60 wt %)/glycerol (10 wt %) system. As shown in Figure 2b, L-L phase separation occurred in this solution. The binodal temperature and the crystallization temperature were 120 and 99 °C, respectively. In this case, the solidification of the solution surface did not start just before the termination of crystallization, but instead began some time after termination of crystallization. The time expected for the start of the gradient increase, based on the relative crystallinity value at which the S-L phase separation system showed an increase, is shown by the dotted line. Clearly, the observed time (solid line) is longer than the expected time (dotted line). In the case of L-L phase separation, droplets of polymer-lean phase exist between the spherulites, as shown in Figure 2b. Thus, mutual contact of the spherulites is not likely to occur, because of obstruction by the droplets. This is why solution stiffness increase began later in this system. Therefore, the solidification rate is slower for the L-L phase separation system than for the S-L phase separation system.

## 3. Experimental Section

### 3.1. Materials

PVDF, Mw = 136,000 (Solef 6008; Solvay Advanced Polymers Co., Alpharetta, GA, USA) was used in the membrane preparation. Glycerol triacetate (GTA) and diethyl phthalate (DEP) were used as solvents and glycerol was used as the nonsolvent. These were purchased from Wako Pure Chemical Industries (Osaka, Japan) and used without further purification.

### 3.2. Crystallization Behavior

A DSC (DSC-7, PerkinElmer Inc., Waltham, MA, USA) was used to determine the dynamic crystallization temperature at a constant cooling rate. The mixture of solid polymer and solvent/nonsolvent was sealed in an aluminum DSC pan, melted at 190 °C for 5 min and then cooled at 10 °C/min to 30 °C. The onset of the exothermic peak during the cooling was taken as the crystallization temperature.

The crystallinity of the polymer solution at time *t* after the onset of crystallization *X_c_*(*t*) was evaluated by the following equation:

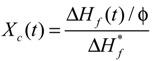
(1)
where 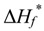 is the melting enthalpy for 100% crystalline PVDF, 104.5 J/g [28], Δ*H_f_*(*t*) is the melting enthalpy of the solution at time *t* after the onset of crystallization, as measured by DSC, and φ is the weight fraction of PVDF in the solution.

### 3.3. Observation of Phase Separation

The polymer-solvent-nonsolvent samples were placed between a pair of microscope cover slips. A Teflon film, 100 µm thick, with a square opening, was inserted between the cover slips. The sample was heated on a hot stage (LK-600PH, Linkam Scientific Instruments Ltd., Surrey, UK) at 150 °C for 2 min and cooled to 30 °C at a controlled rate of 10 °C/min. The temperature of the stage was manipulated by a Linkam L-600A controller (Linkam Scientific Instruments Ltd., Surrey, UK). The phase separation behavior was observed under an optical microscope (BX50, Olympus Corporation, Tokyo, Japan).

### 3.4. Membrane Stiffness Measurement

**Figure 6:**
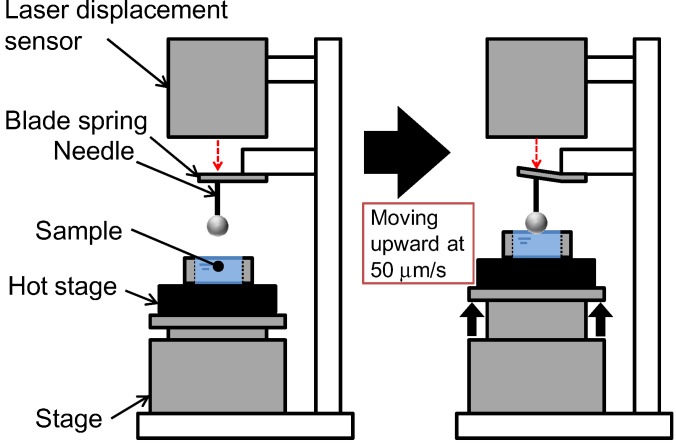
Schematic diagram of stiffness measurement apparatus.

**Figure 7:**
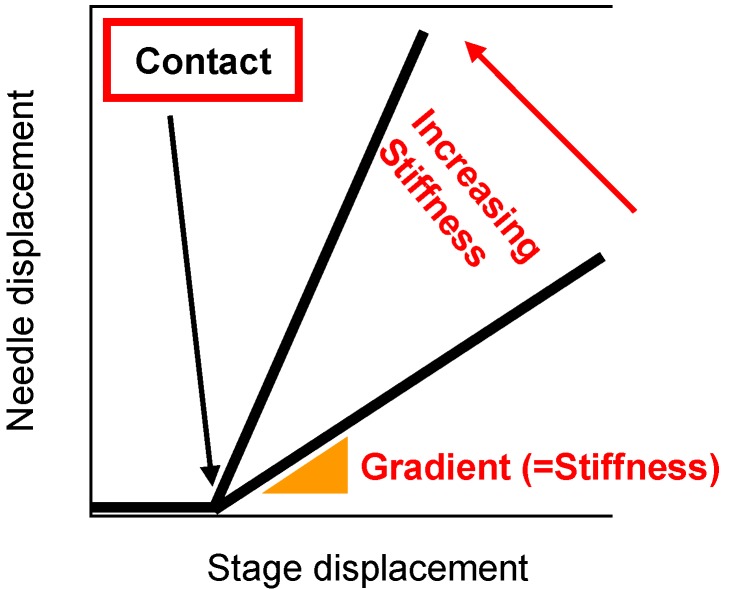
Concept of gradient in plot of needle displacement against stage displacement.

Figure 6 shows a diagram of the stiffness measurement apparatus. This apparatus was the same as that used previously in our laboratory [24]. The hot stage (LK-600PH, Linkam Scientific Instruments Ltd., Surrey, UK) was placed on the movable stage. The hot homogeneous PVDF solution was poured into a washer (thickness 1.4 mm) on a stainless steel sheet (thickness: 0.08 mm) on the hot stage controlled at 150 °C. Then the sample was cooled down by the Linkam L-600A controller to induce phase separation. The stage was moved upward at 50 µm/s and the surface of the polymer solution during phase separation contacted a sphere attached to the tip of a needle. The displacement of a blade spring attached to the needle was measured by a laser displacement sensor. The needle displacement was plotted against stage displacement. When the solution has low stiffness, the gradient of the plot of needle displacement against stage displacement is almost zero, because the sphere attached to the needle goes easily into the solution. On the other hand, when the sample is sufficiently hard, this gradient is close to unity and the stage displacement is equal to the needle displacement. Thus the gradient represents the stiffness of the solution [24]. The concept of this gradient in the plot of the needle displacement against the stage displacement is shown in Figure 7. From each cooling experiment, only one gradient data point was taken, just after the contact between the sphere and the solution surface. Therefore, gradient data at various times after onset of the polymer crystallization were obtained by adjusting the time at which solution cooling began.

## 4. Conclusions

In this study, we quantitatively analyzed the solidification behavior of polymer solutions during the TIPS process by using the apparatus newly developed in our laboratory. In the case of S-L phase separation (polymer crystallization), the stiffness of the solution surface began to increase significantly just before the termination of crystallization. At this time, the spherulites formed by the crystallization come into contact with one another. In contrast, for L-L phase separation, the solidification of the solution surface did not begin just before the termination of crystallization, but instead began some time after the termination of crystallization. This is because the mutual contact of the spherulites is obstructed by the droplets of polymer-lean phase formed by L-L phase separation. Thus, the solidification rate of the L-L phase separation system during phase separation by the TIPS process was slower than that of the S-L phase separation system.
